# Characterization of the radioresponse of human apical papilla-derived cells

**DOI:** 10.1186/scrt43

**Published:** 2011-01-20

**Authors:** Shigehiro Abe, Keiichi Hamada, Satoshi Yamaguchi, Teruo Amagasa, Masahiko Miura

**Affiliations:** 1 Oral Radiation Oncology, Department of Oral Restitution, Tokyo Medical and Dental University, 1-5-45 Yushima, Bunkyo-ku, Tokyo 113-8549, Japan; 2 Maxillofacial Surgery, Department of Maxillofacial Reconstruction and Function, Graduate School of Medical and Dental Sciences, Tokyo Medical and Dental University, 1-5-45 Yushima, Bunkyo-ku, Tokyo 113-8549, Japan

## Abstract

**Background:**

The purpose of this study was to characterize the radiobiological properties of stem/progenitor cells derived from apical papilla-derived cells (APDCs) compared to bulk APDCs.

**Methods:**

APDCs were isolated from freshly extracted human third molars with immature apices. Multipotent spheres, which are thought to contain an enriched population of stem/progenitor cells, were formed from the APDCs, using a neurosphere culture technique. After γ-irradiation, papillary sphere-forming cells (PSFCs) and bulk APDCs were subjected to radiosensitivity and hard tissue-forming assays.

**Results:**

Compared to bulk APDCs, the PSFCs exhibited a radioresistant phenotype and a higher capacity for DNA double strand break repair. Irradiation induced a significant increase in a senescence-like phenotype in both cell types. Neither type of cells exhibited a significant induction of apoptotic changes after 8 Gy of irradiation. Ability to form hard tissue *in vivo *was significantly decreased in PSFCs, but not in APDCs following 4 Gy of irradiation.

**Conclusions:**

We demonstrated for the first time that stem/progenitor cells derived from APDCs exhibit a radioresistant phenotype; however, the hard tissue forming ability *in vivo*, but not bulk APDCs, was significantly reduced after irradiation.

## Introduction

Since the discovery of the dental pulp stem cells (DPSCs) about 10 years ago, several studies have reported various types of DPSCs in mature permanent teeth, developing teeth, and tooth germs [1-5]. It is now a widely held view that DPSCs play a central role in forming structures of teeth [1,2]. We previously reported that apical papilla-derived cells (APDCs) derived from the tip of the apical papilla of human developing third molars with immature apices exhibit high proliferation activity and multilineage differentiation potential, and could, therefore, be an effective source of cells for hard tissue regeneration *in vivo *[6-8]. DPSCs are implied to originate from the cranial neural crest and differentiate not only into the mesenchymal lineage, but also into other lineages [4,6,9]. Neural crest-derived multipotent stem cells have been isolated from many tissues including the skin [10] and bone marrow [11] in rodent models using a sphere formation technique, which enables enrichment of stem/progenitor cells. Lombaert *et al*. succeeded in rescuing salivary gland function by the transplantation of stem cell-like cells, derived from the salispheres formed *in vitro*, into irradiated glands [12].

Dental development is often disturbed (root hypoplasia, etc), for instance after leukemia therapy for children when total body irradiation is combined [13-15]. To the best of our knowledge, the effects of ionizing radiation on human APDCs have never been reported thus far. Therefore, we applied the sphere formation technique to APDCs and eventually identified papillary sphere-forming cells (PSFCs) in human APDCs. We thought that analyzing the radiobiological properties and effects of irradiation would be important on mineralized cell linage differentiation in PSFCs, using the bulk APDCs as a control. We show here that PSFCs are more radioresistant than bulk APDCs and that PSFCs exhibit a significant reduction of the hard tissue formation ability *in vivo*, but not bulk APDCs, following irradiation.

## Materials and methods

### Cell culture

This study was approved by the Institutional Review Board of the Faculty of Dentistry, Tokyo Medical and Dental University. Written informed consent was obtained from all donors. Human impacted third molars freshly extracted for orthodontic or other treatments were obtained from the oral and maxillofacial surgery clinic of Tokyo Medical and Dental University. The APDCs were isolated as previously described [6]. Cryopreserved cells from the second to sixth passages were used for each experiment.

### Papillary sphere culture

We followed a previously reported method with some modifications [16]. Trypsinized APDCs (5 × 10^4 ^cells) were grown in 24-well super-hydrophilic plates (Cellseed, Tokyo, Japan) in serum-free Dulbecco's Minimum Essential Medium (MEM)/F12 (1:1) containing 20 ng/ml of basic fibroblasts growth factor (bFGF) and 20 ng/ml epidermal growth factor (EGF). Primary sphere-forming cells that had been cultured for seven days were used for all experiments. Papillary spheres were fixed in 4% paraformaldehyde, embedded in Optical Cutting Temperature (O.C.T) compound (Sakura Finetechnical Co., Tokyo, Japan), and histologically processed, followed by immunohistochemical analysis.

### Immunofluorescence

Cells were fixed with 4% paraformaldehyde at 4°C and washed twice with Tris-buffered saline with Tween 20 (TBST). The cells and cryosections were incubated in TBST/5% skim milk solution for 30 minutes to prevent non-specific binding of the antibodies. The sides were incubated with an antibody specific for Nestin (1:100) (R&D Systems, Minneapolis, MN, USA), Musashi-1 (1:50) (R&D Systems), osteocalcin (OCN) (1:200) (Takara Bio, Shiga, Japan), α smooth muscle actin (αSMA) (1:200) (Sigma-Aldrich, St. Louis, MO, USA), Tuj-1(βⅢ tubulin) (1:100) (Chemicon, Temecula, CA, USA), microtubule-associated protein-2 (MAP-2) (1:100) (Sigma), or γH2AX (1:500) (Upstate Cell Signaling, Lake Placid, NY, USA) at 37°C for one hour. After washing in phosphate-buffered saline (PBS), the cells were incubated with anti-mouse IgG conjugated with Alexa Fluor 488 (Invitrogen, Carlsbad, CA, USA) and anti-goat IgG conjugated Alexa 594 (Invitrogen) at room temperature for 30 minutes.

### Differentiation of PSFCs

For mineralized cell differentiation, several spheres were plated in chamber slides (BD Bioscience, San Diego, CA) and cultured in condition medium, αMEM supplemented with 10% fetal bovine serum (FBS), for seven days. The medium was changed to αMEM supplemented with 10% FBS, 0.2 mM ascorbic acid, 5 mM β-glycerophosphate and 100 nM dexametasone, and cultured for two weeks, followed by another one-week culture with the same medium plus 10 mM β-glycerophosphate. To evaluate the mineralized matrix, cells were fixed in methanol for 10 minutes and stained with 1% Alizarin red S (Wako Pure Chemical Industries, Osaka, Japan). For adipogenic differentiation, several spheres were cultured as described above. The medium was then changed to adipogenic promoting medium [17] and cultured for three weeks. To identify the adipocytes, cells were stained with Oil red O (Sigma Aldrich, St. Louis, MO, USA). For myogenic differentiation, several spheres were cultured as described above, followed by culture in high-glucose DMEM supplemented with 10% FBS and 10 ng/ml of transforming growth factor-β1 (TGF-β1) (R&D Systems, Minneapolis, MN, USA) for 10 days. Cells were then immunostained with anti-αSMA antibody as described below.

### *In vivo* hard tissue-forming assay

The APDCs and PSFCs were seeded at a density of 5 × 10^4 ^cells into porous hydroxyapatite (HA) scaffolds of Cell Yard™ (Pentax, Tokyo, Japan). Cells were irradiated with 0 or 4 Gy and differentiated into mineralized cells using the protocol described above. Previously described transplantation methods were then employed [7] with some modifications. The cells with HA scaffolds were implanted in subcutaneous pouches of the dorsum of four- to seven-week old male KSN nude mice (Sankyo Laboratory, Tokyo, Japan). After 12 weeks, the implanted tissues were removed and histological preparations were made as described previously [7]. For the quantitative analysis of hard tissue formation, five sections were randomly selected from consecutive sections in each sample. The volume of newly formed hard tissue in the porous area was quantified using a Photoshop software (Adobe, San Jose, CA, USA), and the area was calculated as the percentage of regenerated hard tissue in the porous area.

### Colony-forming assay and irradiation

Cells derived from APDCs or PSFCs were enzymatically dissociated with trypsin-EDTA solution and mechanically dissociated with a Pasteur pipette. An appropriate number of cells were plated in six-well tissue culture dishes. On the day after seeding, cells were exposed to γ-rays (0, 2, 4, and 6 Gy) using a ^60^Co γ-ray therapeutic machine (TOSHIBA, Tokyo, Japan) at a dose rate of 0.62 Gy/minute. After 14 days of incubation, the cells were fixed with 10% formalin, and stained with crystal-violet. Colonies containing more than 50 cells were counted and the surviving fractions were determined. Three independent experiments were performed for each sample.

### Double strand break (DSB) repair kinetics of APDCs and PSFCs

Cells grown on eight-well chamber slides (BD Bioscience, San Jose, CA, USA) were irradiated as above with 0 or 8 Gy irradiation. γ-H2AX immunofluorescence analysis was performed either 30 minutes or 24 hours after irradiation. For the quantitative analysis, we counted γ-H2AX foci in the nuclei of the samples. One hundred randomly selected cells were counted for each sample.

### Senescence-associated β-galactosidase assay

Exponentially growing cells were incubated for three days after 4 Gy of irradiation. The resultant cells were used for senescence-associated β-galactosidase (SA-β-gal) staining, using an SA-β-gal Staining Kit (Sigma) according to the manufacturer's instructions [18]. For quantitative analysis, the percentages of SA-β-gal-positive and enlarged cells were determined by counting at least 200 cells from randomly selected fields in each sample.

### Detection of apoptotic cells

Cells were irradiated with 0 or 8 Gy and assayed using terminal deoxynucleotidyl transferase-mediated deoxyuridine triphosphate nick-end labeling (TUNEL). Twenty-four hours after irradiation, TUNEL assay was performed using an *In Situ *Cell Death Detection Kit (Roche Diagnostic/Boehringer Mannheim Co., Indianapolis, IN, USA) according to the manufacturer's instructions and analyzed with a BX51 fluorescence microscope (Olympus, Tokyo, Japan). DNA was simultaneously stained with DAPI. APDCs were treated with 2 mM H_2_O_2 _(Wako Pure Chemical Co., Ltd., Osaka, Japan) for one hour, prepared for TUNEL assay after the treatment, and used as a positive control.

### Statistical analysis

Mean values were compared using a Student's *t*-test. *P-*values < 0.05 were considered statistically significant.

## Results

### Macroscopic appearance of human apical pulp tissues and characteristics of PSFCs derived from APDCs

**Figure 1:**
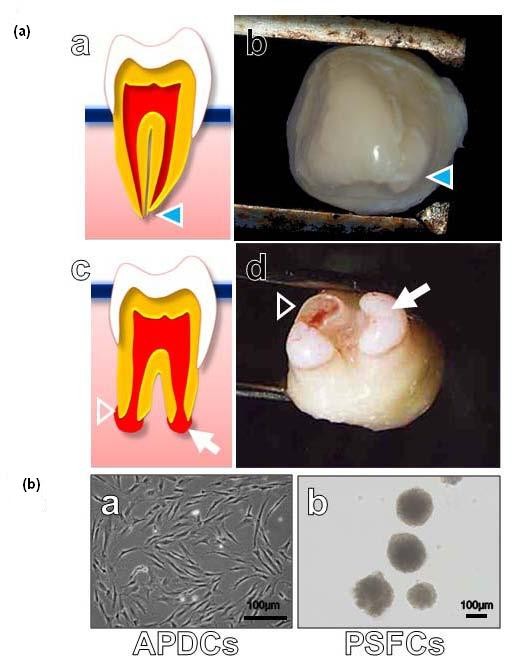
**Macroscopic appearance of human apical papilla tissues and characteristics of papillary sphere-forming cells (PSFCs)**. **(a) **Photographs of human apical papilla tissues attaching to the teeth. (a, b) Human tooth with mature apex. Arrowhead indicates mature root apex. (c, d) Human tooth with immature apex. Arrow indicates apical papilla tissue not framed in by dentin. Arrowhead indicates formation of the root with immature apex. **(b) **Morphology of cultured apical papilla-derived cells (APDCs) *in vitro*. (a) Exponentially growing APDCs under monolayer culture. (b) Papillary sphere-forming cells (PSFCs).

**Figure 2:**
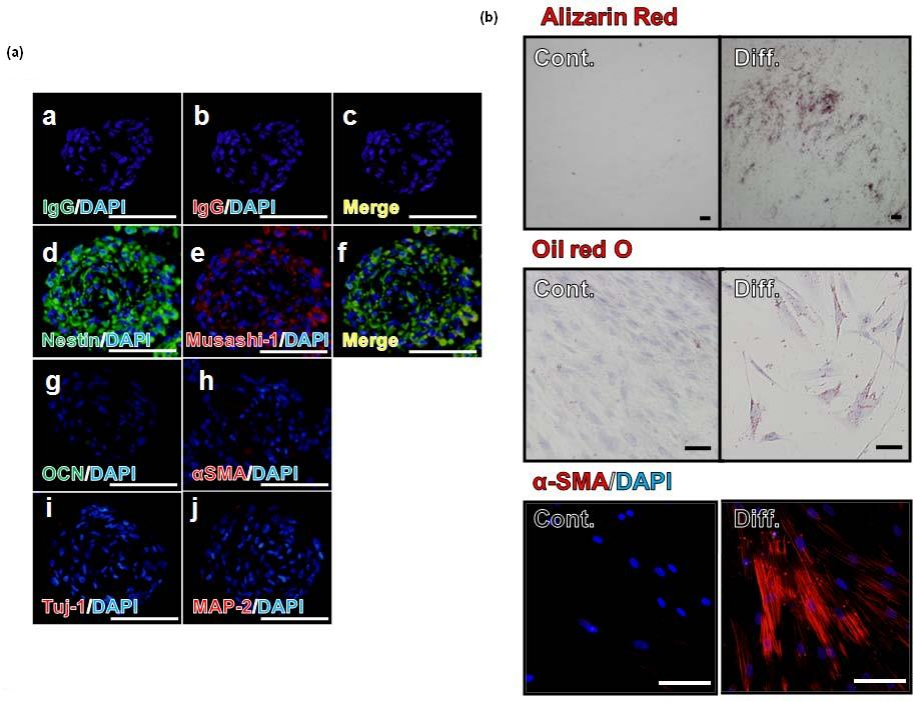
**Differentiation markers and multipotent differentiation capacity in PSFCs**. **(a) **Differentiation markers of PSFCs. Negative control was incubated with only secondary antibodies (a-c). Nestin (d), Musashi-1 (f), osteocalcin (OCN)(g), αSMA (h), Tuj-1 (i) and MAP-2 (j). Scale: 100 μm. **(b) **Multipotent differentiation capacity of PSFCs. PSFCs were differentiated under specific culture conditions suitable for differentiation into each cell type. Identification of differentiation: Alizarin red staining for mineralized cells; Oil red O staining for adipocytes; αSMA immunostaining for myocytes. Cont, before differentiation; Diff,: after differentiation. Scale: 100 μm.

The human tooth with an immature apex is a developing organ. The stem cells near the root apex contribute to the formation of the complete root [5,6]. The APDCs were derived from the soft tissue of the tip of the apical papilla (Figure 1Ac, d), which consists of a mixture of cell types, including active odontoblasts and fibroblasts. PSFCs were further isolated from APDCs using a neurosphere technique, as described in the Introduction section (Figure 1B). The isolated PSFCs expressed neural stem cell or neural crest stem cell-specific markers, such as Nestin and Musashi-1, but did not express lineage markers such as osteocalcin (OCN) (mineralized cell marker), αSMA (smooth muscle cells marker), Tuj-1, or MAP-2 (neural cell marker) (Figure 2A). The cells also exhibited multipotency: they were capable of differentiating into mineralized cells (stained with Alizarin red), adipocytes (stained with Oil red O), and myocytes (immunostained with anti-α SMA antibody) under the appropriate culture conditions (Figure 2B). These findings suggested that PSFCs consist of a population that is enriched in stem/progenitor cells and that the majority of PSFCs are in an undifferentiated state. We thus took advantage of this technique to analyze the radiobiological properties of stem/progenitor cells derived from APDCs, by comparing them with those of bulk APDCs.

### PSFCs exhibit a radioresistant phenotype compared to APDCs

**Figure 3:**
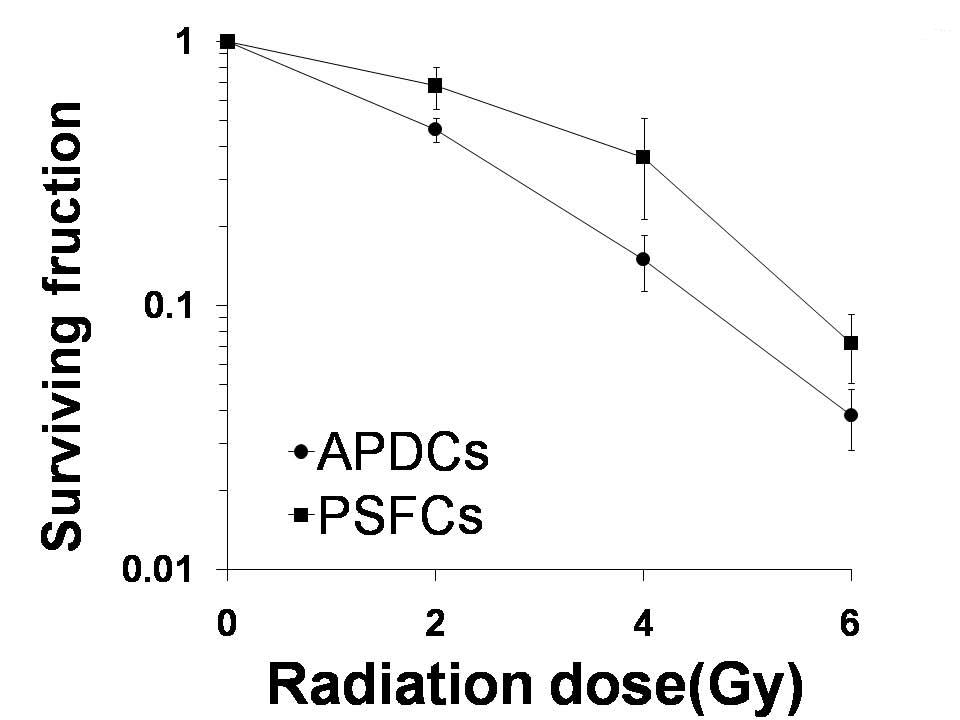
**Dose-cell survival curves following γ-irradiation obtained by colony-forming assay**. Results are expressed as means ± SE of four different samples.

We first examined the intrinsic radiosensitivity of exponentially growing APDCs and PSFCs by colony-forming assay (Figure 3). To avoid complications from sphere formation-associated factors that may influence radiosensitivity [19], cells were irradiated after trypsinization of spheres. The results clearly showed that PSFCs are more radioresistant than bulk APDCs.

**Figure 4:**
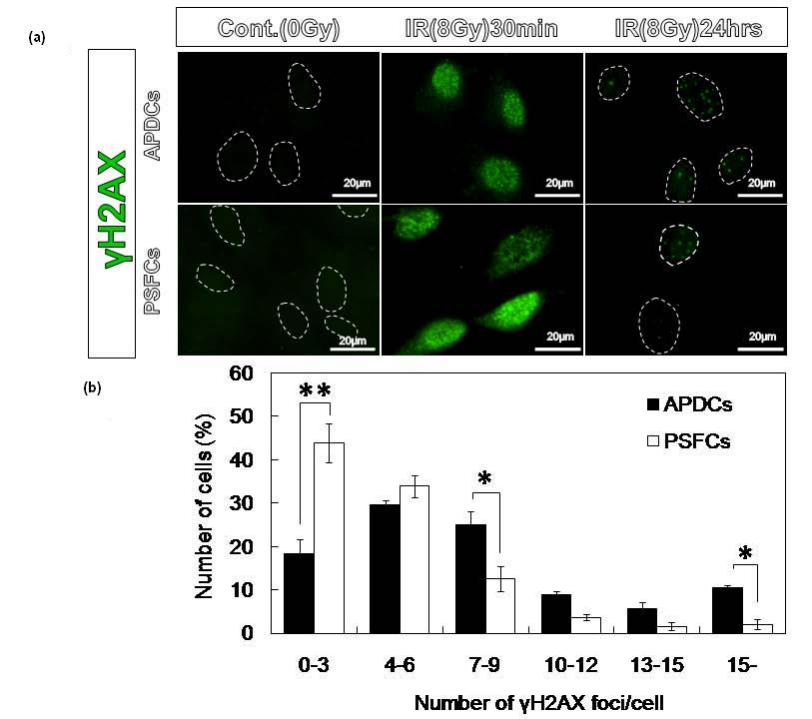
**Double strand break (DSB) repair kinetics in APDCs and PSFCs following irradiation**. **(a) **Immunofluorescent foci of γ-H2AX. At the indicated times after 8 Gy of irradiation, cells were fixed and prepared for immunofluorescence staining. **(b) **Histograms of the number of γ-H2AX foci per nucleus 24 h after irradiation. Results are expressed as means ± SE of three different samples. *P < 0.05, **P < 0.001. Scale: 20 μm.

We next examined the kinetics of DSB repair, a pathway that is of critical importance in determining cellular radiosensitivity [20]. For this purpose, we quantified the number of immunofluorescent foci of histone H2AX phosphorylated at Ser 139 (γ-H2AX), which are formed in close proximity to DSB sites. Because γ-H2AX disappears after DSB repair is completed, the rate at which γ-H2AX disappears following irradiation reflects DSB repair activity. Therefore, we compared DSB repair activity in APDCs and PSFCs using an immunohistochemical method specific for γ-H2AX. Thirty minutes after irradiation, many γ-H2AX foci were detected in the nuclei of both APDCs and PSFCs; 24 h after irradiation, fewer γ-H2AX foci were detected in both cell types (Figure 4A). We quantitatively analyzed the DSB repair by counting the γ-H2AX foci 24 h after irradiation and the histograms are shown in Figure 4B. The histogram in APDCs shifted to the right side compared to that in PSFCs, indicating that the DSB repair capacity is higher in PSFCs than in APDCs. The results are thus consistent with intrinsic radiosensitivity determined by colony-forming assay.

### Irradiation significantly induces senescence-like phenotype, but not apoptotic changes in PSFCs

**Figure 5:**
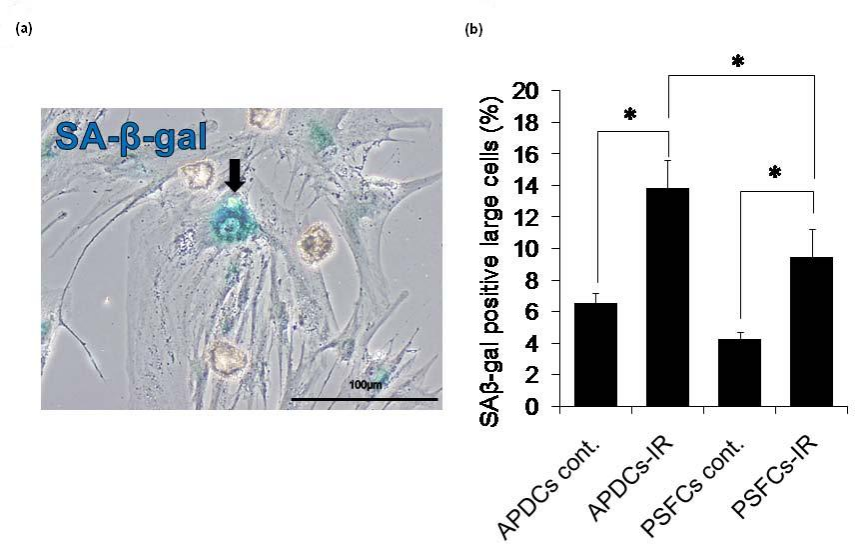
**Induction of the senescence-like phenotype in APDCs and PSFCs after irradiation**. **(a) **A typical example of senescence-like cells. **(b) **Quantitative analysis of the induction of the senescence-like phenotype in APDCs and PSFCs after irradiation. For quantitative analysis, senescence-associated -β-galactosidase-positive and enlarged cells were considered to be senescent cells. The percentages of senescent cells three days after 4 Gy of irradiation were determined in APDCs and PSFCs. At least 200 cells were counted in each group. Results are expressed as means ± SE of three independent samples. *, *P *< 0.05. Scale: 100 μm.

**Figure 6:**
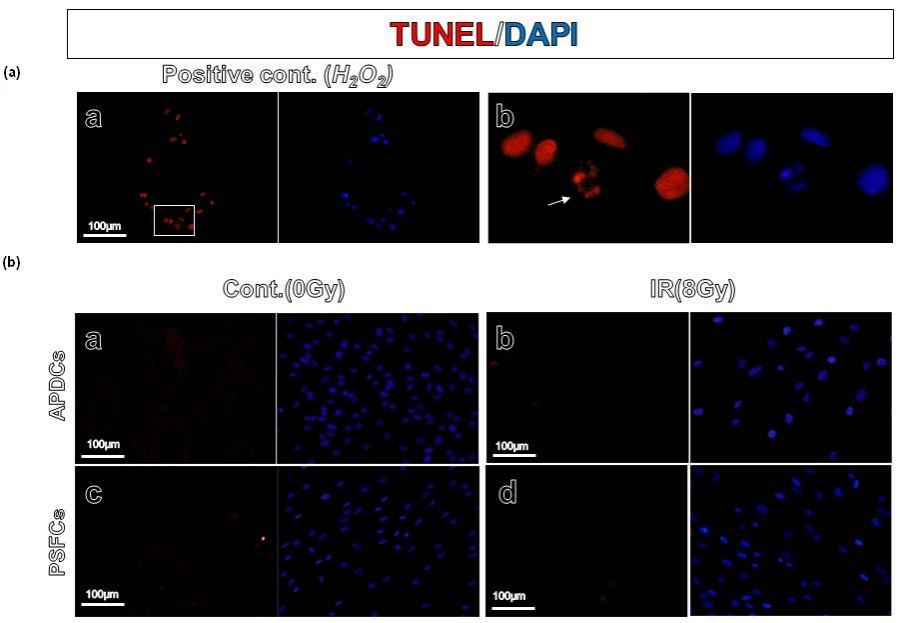
**Apoptotic changes in APDCs and PSFCs following irradiation**. **(a) **A typical example of apoptosis in APDCs. As a positive control (a), APDCs were treated with 2 mM H_2_O_2 _and terminal deoxynucleotidyl transferase-mediated deoxyuridine triphosphate nick-end labeling (TUNEL) assay was performed one hour after the treatment. (b) A magnified field of the square within panel a: the arrow indicates nuclear fragmentation. **(b) **Apoptotic changes in APDCs and PSFCs following irradiation. TUNEL assay was performed 24 h after 8 Gy of irradiation. Nuclear staining was done with DAPI. Scale: 100 μm.

**Figure 7:**
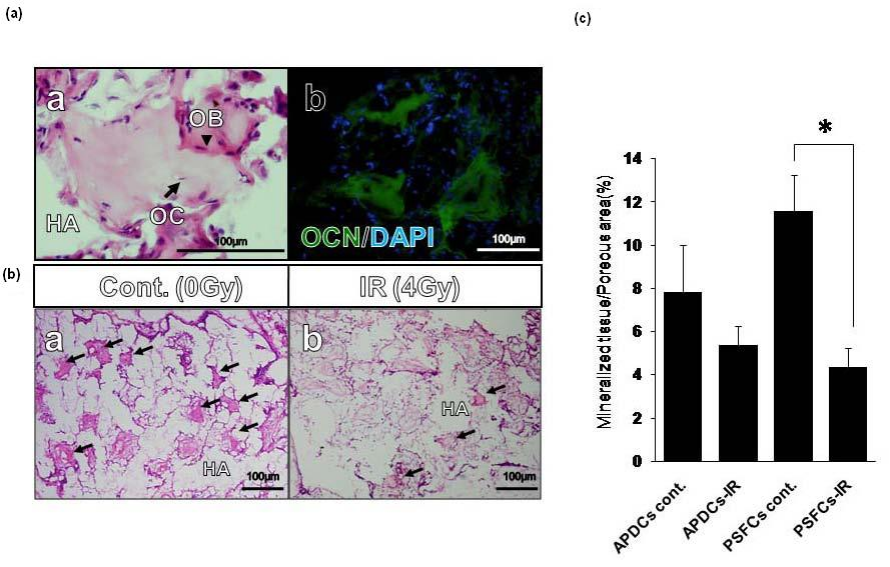
**Effect of irradiation on hard tissue-forming capacity *in vivo *in APDCs and PSFCs**. **(a) **A typical example of hard tissue generation *in vivo*. (a) Generated bone-like hard tissue. Osteocyte-like cells (OC) embedded in the mineralized matrix and osteoblastic cells (OB) lining the surface of the hard tissue. HA, hydroxyapatite. (b) Immunofluorescence staining for osteocalcin (OCN). green: OCN; blue: nuclei. Scale: 100 μm. **(b) **A typical example of hard tissue generation *in vivo *in non-irradiated and irradiated cells. HA, hydroxyapatite. **(c) **Quantitative analysis of the generated hard tissue area in three independent samples. Results are expressed as means ± SE of five pieces of sections in each sample. *P < 0.05.

We next performed senescence-associated β-galactosidase (SA-β-gal) staining after irradiation. In some normal cells, a senescence-like phenotype, rather than apoptosis, is associated with radiosensitivity [21-23]. We found that the percentages of characteristic phenotypes of senescence (SA-β-gal-positive, flattened and enlarged morphology; (Figure 5A), significantly increased in both APDCs and PSFCs following 4 Gy irradiation (Figure 5B). The induction was significantly lower in PSFCs. Similar results were also obtained when cells were irradiated at 8 Gy (data not shown). On the other hand, irradiation caused a negligible level of apoptotic changes in both cell types, as determined by immunohistochemical analysis using TUNEL method, even after 8 Gy of irradiation (Figure 6A). Neither nuclear fragmentation nor apoptotic bodies were observed 24 h after irradiation (Figure 7B).

### *In vivo *hard tissue generation

To investigate the effects of irradiation on the potential capacity of PSFCs to form hard tissue *in vivo*, composites of cells and HA were implanted subcutaneously into immunocompromised mice. By 12 weeks after implantation, APDCs and PSFCs had formed ectopic hard tissues on the border of the porous HA. The hard tissues contained osteocyte-like cells embedded within a calcified matrix, and osteoblast-like cells were observed along the surface of the lining on the bone surface (Figure 7Aa). Furthermore, hard tissues generated by the implants expressed osteocalcin (OCN), which is a mature mineralized cell marker (Figure 7Ab). Quantitative analysis revealed that PSFCs tended to possess a greater ability to form mineralized tissues than APDCs, and that this ability was significantly inhibited after irradiation; this could be owing to a reduced stem cell-associated activity (Figures 7B, C).

## Discussion

The stem/progenitor cells that are present in APDCs derived from the tip of the apical papilla play an essential role in root formation of developing teeth [13-16]. We, therefore, reasoned that the radiobiological properties of the PSFCs derived from APDCs would help us to understand the disturbance of dental development such as root hypoplasia that occurs in pediatric patients for instance after leukemia therapy combined with total body irradiation [13-15].

Tissue regeneration is impaired after DNA damage; the shrinkage of the stem cell pool has been implicated as a mechanism underlying this diminished capacity [24-26]. This mechanism could operate by induction of apoptosis, senescence, or abnormal differentiation by stressed stem cells. Irradiation can lead to apoptosis or cellular senescence in hematopoietic stem cells and mesenchymal stem cells, resulting in compromised self-renewal of stem cells and shrinkage of the pool [27,28]. Inhibition of differentiation after DNA damage has also been observed in mouse satellite cells derived from C2C12 myoblasts [29,30]. Schönmeyr *et al*. found that irradiation reduced alkaline phosphatase (ALP) activity and the expression of osteocalcin in differentiating MSCs and inhibited differentiation into osteoblasts [28]. Recently, Inomata *et al*. reported that the DNA-damage response triggers melanocyte stem cell differentiation into premature melanocytes in the niche, rather than inducing their apoptosis or senescence [31]. The idea that enhancement of differentiation contributes to the stem cell depletion is an interesting concept.

In general, active mineralized cells have high expression of ALP and only fully mature mineralized cells can produce a matrix that can be subsequently mineralized *in vitro *and *in vivo *[32]. We found no evidence that irradiation enhances ALP activity *in vitro *in an appropriate differentiating condition (data not shown). Therefore, our findings that PSFCs exhibited a significant reduction of the hard tissue generation capacity *in vivo *raise two possibilities: 1) irradiation alters the mineralized cell linage differentiation program, leading to the inhibition of differentiation in stem/progenitor cells; and/or 2) irradiation induces massive stem/progenitor cell killing, resulting in a reduction in the number of functional differentiated cells without affecting the differentiation process. Considering that PSFCs were more radioresistant than APDCs, and that apoptosis induction was negligible in both cell types, the current findings favor the former possibility. Compared to PSFCs, APDCs are thought to contain larger numbers of differentiated cells before irradiation, ensuring that the mineralizing process by itself is not much affected, at least by irradiation at doses used in this study.

During the *in vivo *development of dental roots, additional environmental complexities exist. Root dentin is normally formed during the root development via interactions of epithelial cells (Hertwig's epithelial root sheath cells: HERSCs) and dental papilla cells [33]; therefore, dentin is unlikely to develop under conditions in which only APDCs are present. However, other groups reported [3,34] that stem-like cells derived from dental pulp have a bone-like hard tissue-forming ability *in vivo*, even in the absence of HERSCs. We assumed that this ability of APDCs, determined using the relatively simple and widely used differentiation method for hard tissue formation, would correlate with the *in vivo *dentin-forming ability. To maintain and regulate the stem cells, the tissue microenvironment (niche) also plays an important role *in vivo*; however, the niche of dental papilla stem cells has not yet been carefully characterized, and the important interactions are not yet known in detail. *In vivo *studies including such factors will be required in order for us to understand the effects of irradiation on root dysplasia.

## Conclusions

We demonstrated for the first time that stem/progenitor cells derived from APDCs exhibit a radioresistant phenotype and a significant reduction of hard tissue forming ability *in vivo *but not bulk APDCs, following irradiation.

## Abbreviations

αSMA: α smooth muscle actin; γ-H2AX: phosphorylated histone H2AX; ALP: alkaline phosphatase; APDCs: apical papilla-derived cells; DPSCs: dental pulp stem cells; DSB: double strand break; FBS: fetal bovine serum; HA: hydroxyapatirte; MAP-2: microtubule-associated protein-2; OCN: ostecalcin; PBS: phosphate buffered saline; PSFCs: papillary sphere-forming cells; SA-β-Gal: senescence-associated β galactosidase; TBST: Tris-buffered saline with Tween 20; TUNEL: terminal deoxynucleotidyl transferase-mediated deoxyuridine triphosphate nick-end labeling.

## Competing interests

The authors declare that they have no competing interests.

## Authors' contributions

SA carried out most of the study and participated in its design. KH, SY, and TA participated in the study design and data discussion. MM jointly conceived of the study and participated in its design and drafted the manuscript. All authors read and approved the final manuscript.
